# The Disassociation of the A20/HSP90 Complex *via* Downregulation of HSP90 Restores the Effect of A20 Enhancing the Sensitivity of Hepatocellular Carcinoma Cells to Molecular Targeted Agents

**DOI:** 10.3389/fonc.2021.804412

**Published:** 2021-12-15

**Authors:** Li-jun Shen, Hui-wei Sun, Yan-yao Chai, Qi-yu Jiang, Jian Zhang, Wen-ming Li, Shao-jie Xin

**Affiliations:** ^1^ Medical School of Chinese People’s Liberation Army (PLA), Chinese People’s Liberation Army (PLA) General Hospital, Beijing, China; ^2^ Division 8, Department of Hepatology, Senior Department of Hepatology, The Fifth Medical Center of Chinese People’s Liberation Army (PLA) General Hospital, Beijing, China; ^3^ Senior Department of Infectious Disease, Institute of Infectious Disease, The Fifth Medical Center of Chinese People’s Liberation Army (PLA) General Hospital, Beijing, China; ^4^ Department of Patient Management, The Fifth Medical Center of Chinese People’s Liberation Army (PLA) General Hospital, Beijing, China; ^5^ Department of Emergency Medicine, Handan Central Hospital, Handan, Hebei Province, China; ^6^ Division 6, Department of Hepatology, Senior Department of Hepatology, The Fifth Medical Center of Chinese People’s Liberation Army (PLA) General Hospital, Beijing, China

**Keywords:** A20, heat-shock protein 90, advanced hepatocellular carcinoma, molecular targeted agents, drug resistance

## Abstract

NF-κB (nuclear factor κB) is a regulator of hepatocellular cancer (HCC)-related inflammation and enhances HCC cells’ resistance to antitumor therapies by promoting cell survival and anti-apoptosis processes. In the present work, we demonstrate that A20, a dominant-negative regulator of NF-κB, forms a complex with HSP90 (heat-shock protein 90) and causes the disassociation of the A20/HSP90 complex *via* downregulation of HSP90. This process restores the antitumor activation of A20. In clinical specimens, the expression level of A20 did not relate with the outcome in patients receiving sorafenib; however, high levels of HSP90 were associated with poor outcomes in these patients. A20 interacted with and formed complexes with HSP90. Knockdown of HSP90 and treatment with an HSP90 inhibitor disassociated the A20/HSP90 complex. Overexpression of A20 alone did not affect HCC cells. Downregulation of HSP90 combined with A20 overexpression restored the effect of A20. Overexpression of A20 repressed the expression of pro-survival and anti-apoptosis-related factors and enhanced HCC cells’ sensitivity to sorafenib. These results suggest that interactions with HSP90 could be potential mechanisms of A20 inactivation and disassociation of the A20/HSP90 complex and could serve as a novel strategy for HCC treatment.

## Introduction

Hepatocellular carcinoma (HCC) is a significant challenge to China’s public health system because of the high infection rates with hepatitis virus (1). These patients are at significant risk of progressing to HCC, and most patients are in the advanced stage of disease at the initial diagnosis (2, 3). Unfortunately, outcomes for patients with advanced HCC are unsatisfactory because of resistance to radiotherapy and multi-drug resistance (4–6). For these reasons, molecularly targeted agents are ever more critical for treating advanced HCC (7, 8). Although targeted molecular drugs represented by sorafenib in several clinical trials were shown to prolong survival and improve quality of life, molecular targeted therapy is also associated with many problems: (1) sorafenib treatment produces drug resistance and severe side effects (9); (2) in addition to sorafenib, new molecular targeted drugs such as lenvatinib, regorafenib, and cabozantinib have been marketed (10–12). Although these drugs are considered superior to sorafenib, they have similar structural features and the same chemical structure as sorafenib (1-(4-(pyridin-4-yloxy)phenyl)urea). For these reasons, lenvatinib, regorafenib, and cabozantinib may not be able to overcome all the insufficiencies of sorafenib. Therefore, it has become essential to research and explore the resistance mechanism of HCC cells to molecularly targeted drugs to develop new and more effective treatment strategies to achieve the same and better anti-tumor effects with smaller doses of drugs.

Several lines of evidence suggest that NF-κB is activated by upstream signals pathways to enhance anti-apoptosis and pro-survival of cancerous cells by mediating expression genes such as Bcl-2, cIAPs (cellular inhibitor of apoptosis), and survivin (13, 14). Kang et al. found that blocking the activation of the Notch-1/NF-κB pathway using small molecular inhibitors enhanced the sensitivity of cancer cells to ionizing radiation (IR) (15). This evidence suggests that inhibition of NF-κB activation may enhance HCC cells’ sensitivity to antitumor therapies, including molecular targeting agents (16, 17). The master suppressor of NF-κB A20 is tumor necrosis factor-alpha-induced protein 3 (18, 19) that is thought to be a tumor suppressor (20). Overexpression of A20 in HCC cells enhanced HCC cells’ sensitivity to radiotherapies *via* suppressing the expression of NF-κB downstream pro-survival and anti-apoptosis-related factors (6). In the present work, unexpectedly, we were first to show that overexpression and knockdown of A20 did not affect the expression of NF-κB downstream pro-survival and anti-apoptosis-related factors in HCC cells. Mechanistically, A20 formed a complex with HSP90, and the disassociation of the A20/HSP90 complex *via* downregulation of HSP90 restored the effect of A20 enhancing the sensitivity of HCC cells to sorafenib.

## Material And Methods

### Clinical Specimens and Ethics Statement

The clinical specimens (52 paired HCC and non-tumor tissues) used in the present work were described in our previous publications (21). The ethics committee approved the use of human-related materials from the Fifth Medical Center, General Hospital of the Chinese PLA. We obtained written consent from the patients.

### Cell Lines and Agents

The hepatic cell lines, including L-02 (hepatic non-tumor cell line) and HCC cell lines (MHCC97-H, MHCC97-L, HepG2, Huh-7, SMMC-7721, and BEL7402), were described in our previous publications (22, 23). The cells were cultured in DMEM with 10% FBS. The molecular targeting agents, sorafenib (Cat. No.: S7397), regorafenib (Cat. No.: S1178), lenvatinib (Cat. No.: S1164), and cabozantinib (Cat. No.: S1119) were purchased from Selleck Corporation, Houston, Texas, USA. The inhibitor of HSP90 BIIB021 was purchased from the Selleck Corporation. The formulations of these agents used in the cellular and animal experiments were prepared by the methods described by Ma et al., Wang et al., and Zhou et al. (24–26). The siRNA (sequence: GCAGCAAAGUGGCGUAUUA) (27) of HSP90 and the full-length sequence of A20 were cloned and prepared as lentivirus particles.

### qPCR and Survival Analysis

Total RNA samples were extracted using a PARISTM Kit (Thermo Fisher Scientific, Waltham, MA, USA) from HCC cells and tumor tissues. They were reverse transcribed using a Multiscribe™ Reverse Transcriptase (Thermo Fisher Scientific) agent following the manufacturer’s instructions. The quantitative polymerase chain reaction (qPCR) was performed following the manufacturer’s instructions and previously described methods (28, 29). mRNA levels of β-actin (loading control) were measured as the loading control. Primers used in qPCR experiments are listed: (1) HSP90 (HSP90AA1): Forward Sequence, 5’-TCTGCCTCTGGTGA TGAGATGG-3’; Reverse Sequence, 5’-CGTTCCACAAAGGCTGAGTTAGC-3’; (2) A20 (TNFAIP3): Forward Sequence, 5’-CTCAACTGGTGTCGAGAAGTCC-3’; Reverse Sequence, 5’-TTCCTTGAGCGTGCTGAACAGC-3’; (3) BCL2: Forward Sequence, 5’-ATCGCCCTGT GGATGACTGAGT-3’; Reverse Sequence, 5’-GCCAGGAGAAATCAAACAGAGGC-3’; (4) Survivin (BIRC5) Forward Sequence, 5’-CCACTGAGAACGAGCCAGACTT-3’; Reverse Sequence, 5’-GTATTACAGGCGTAAGCCACCG-3’; (5) β-Actin (ACTB) Forward Sequence, 5’-CACCATTGGCAATGAGCGGTTC-3’; Reverse Sequence, 5’-AGGTCTTTGCGGATGT CCACGT-3’. In HCC tissue specimens, according to the median A20 and HSP90 mRNA levels, the patients were divided into the A20 high expression group, the A20 low expression group, the HSP90 high expression group, and the HSP90 low expression group. Survival analysis was performed following the methods described by Feng et al. (21).

### The Immunoprecipitation and Western Blot

MHCC97-H cells were cultured and harvested for immunoprecipitation according to our previous publications (30–32). Briefly, the MHCC97-H cells were harvested for immunoprecipitation and western blotting. The HSP90/A20 complexes were separated using mouse anti-human HSP90 antibody (αm-HSP90) (IP: HSP90), and the HSP90 and A20 in the complexes were probed using by rabbit anti-human HSP90 and A20 antibody (αR-HSP90) (IP: HSP90; IB: HSP90 and IP: HSP90; IB: A20). The antibodies were purchased from Abcam Corporation, UK, and Santa Cruz Corporation, USA. Western blotting was performed following the methods described by Yang et al. (33). MHCC97-H cells were transfected with control and siA20; MHCC97-L cells were transfected with control and A20 vectors. The cells were harvested for western blotting, and the expression levels of pro-survival and anti-apoptosis-related factors were examined using antibodies.

### Cell Survival Analysis

Cell survival analysis was performed to determine the antitumor effects of molecular targeting agents on HCC cells. HCC cells were cultured using DMEM with 10% FBS and treated with indicated concentrations of molecular targeting agents. For the MTT experiments, HCC cells were harvested and seeded into 96-well plates (about 8000 cells per well). Then, cells were treated with the indicated concentration of the molecular targeting agents (10 μmol/L, 3 μmol/L, 1 μmol/L, 0.3 μmol/L, 0.1 μmol/L, and 0.03 μmol/L) for 48 h. After treatment, cells were treated with 50 mmol/L MTT for 4 h, and the cells were harvested. We determined cell numbers using the optical density (OD) values at 490 nm. The inhibitor rates of molecular targeting agents on HCC cells were calculated according to the OD values at 490 nm, and the IC_50_ values were calculated based on the inhibitory rates (34, 35). For colony formation, the HCC cells were transfected with plasmids, treated with agents, and seeded onto 6-well plates (2000 cells per well). After 3-4 weeks’ growth, HCC cells formed colonies. We used absolute ethanol to fix the colonies formed by HCC cells in 6-well plates, stained the colonies with 0.5% W/V crystal violet staining solution, and obtained pictures to quantitatively analyze the images according to published methods (36, 37).

### The *In Vivo* Tumor Model and Ethics Statement

The *in vivo* growth of HCC cells was measured using a nude mice model (38, 39). All animal experiments were approved by the Institutional Animal Care and Use Committee of the Chinese PLA General Hospital, China. All animal experiments (n = 10 for each group and animals were randomly divided into the groups) were performed according to the UK Animals (Scientific Procedures Act, 1986) and the associated guidelines. For the subcutaneous tumor model, MHCC97-H cells were injected into the nude mice’s subcutaneous position to form tumor tissues. Mice then received sorafenib and BIIB021 *via* oral administration following the methods described previously (40). The tumor tissues were harvested, and the tumor volumes and weights were measured (41). Expression levels of A20, HSP90, survivin, and BCL-2 in tumor tissues were measured using qPCR.

### The Intra-Hepatic Tumor Model

The *in vivo* growth of HCC cells was further examined using the intra-hepatic tumor model (40). MHCC97-H cells were cultured and transplanted into the nude mice’s livers as described (40). Mice were treated with sorafenib and BIIB021 *via* oral administration as described (40). The intrahepatic growth of MHCC97-H cells in nude mice was measured by (1) mocroPET; (2) radio-activation of the liver to blood; and (3) the quantitative analysis of the images of livers with nodules (40). We used microdissection and other methods to separate liver nodules. The expression levels of A20, HSP90, survivin, and BCL-2 were measured using qPCR (42).

### Statistical Analysis

In the presence work, the biological and technical replicates have been carried out for all experiments and the results were from triple repeats with similar results. All statistical significance analyses were performed using SPSS 9.0 statistical software (IBM Corporation, Armonk, NY, USA). The IC_50_ values were calculated using Origin software (Origin 6.1; OriginLab Corporation, Northampton, MA, USA). Statistical significance was analyzed using Bonferroni correction with two-way ANOVA, and paired samples were tested using the paired-sample t-test (SPSS Statistical Software v16.0; SPSS Inc., Chicago, IL, USA).

## Results

### Overexpression and Knockdown of A20 Did Not Affect HCC Cells

**Figure 1:**
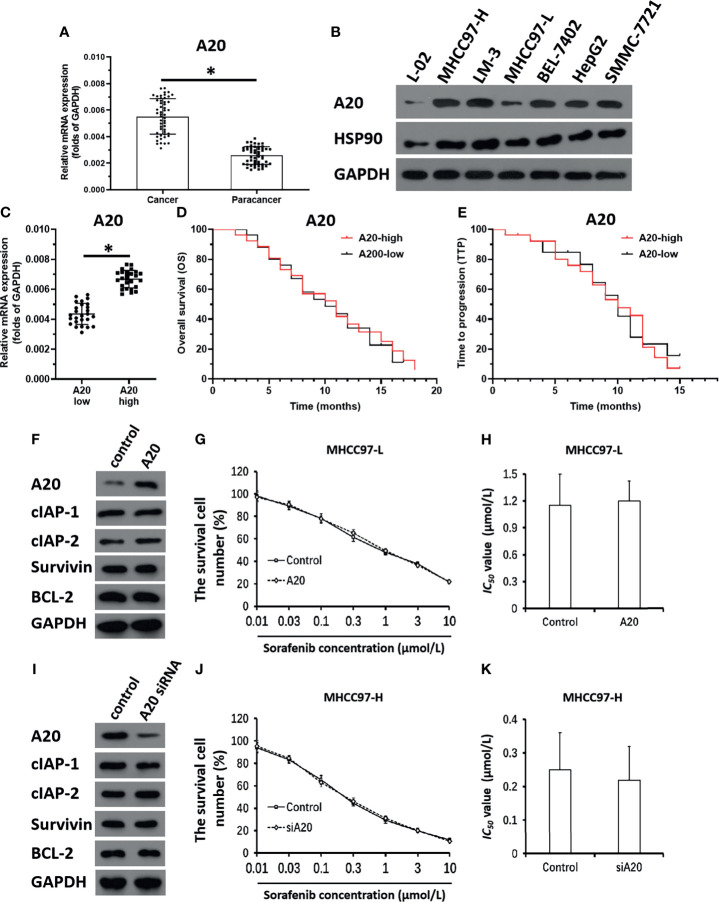
A20 does not affect the sensitivity of HCC cells to molecular targeted agents. **(A)** The expression level of A20 in HCC or the paired non-tumor tissues was examined by qPCR. **(B)** The expression level of A20 in hepatic cell lines was measured by the western blot. **(C)** The expression of A20 in HCC clinical specimens was examined by qPCR and the patients were divided into two groups: A20-high group and A20-low group. **(D, E)** The prognosis of two groups’ patients was revealed by the OS (overall survival) **(D)** or the TTP (time to progress) **(E)**. **(F–H)** MHCC97-L was transfected with control or the A20 vectors. **(F)** The expression level of pro-survival or anti-apoptosis related factors was examined by western blot. **(G, H)** The MHCC97-L cells which were transfected with plasmids were treated with the indicated concentration of Sorafenib and the results were shown as the concentration-effect curve of Sorafenib **(G)** or the *IC_50_* values **(H)**. **(I–K)** MHCC97-H was transfected with control or the A20 siRNA vectors. **(I)** The expression level of pro-survival or anti-apoptosis related factors was examined by western blot. **(J, K)** The MHCC97-H cells which were transfected with plasmids were treated with the indicated concentration of Sorafenib and the results were shown as the concentration-effect curve of Sorafenib **(J)** or the *IC_50_* values **(K)**. *P < 0.05.

To examine the roles of A20 in HCC, expression levels of A20 in clinical specimens were measured. As shown in **Figure 1A**, expression levels of A20 were much higher in HCC tissues than in non-tumor tissues. In hepatic cell lines, expression levels of A20 were much lower in L-02, a non-tumor hepatic cells line, than in HCC cells (**Figure 1B**). Among the HCC cells, the MHCC97-L cells had the lowest expression levels of A20, and the MHCC97-H cells had the highest expression levels (**Figure 1B**). Therefore, MHCC97-H cells were used to knockdown A20, whereas MHCC97-L cells were used to overexpress A20 in subsequent experiments.

The correlation of A20 expression with HCC patients’ outcomes was further examined. In HCC tissue specimens, according to the median of A20 mRNA expression, patients were divided into two groups: the A20 high expression group and the A20 low expression group (**Figure 1C**). The survival analysis of the two groups showed that there was no correlation between expression levels of A20 and outcome patients receiving sorafenib (**Figures 1D, E** and **Table 1**), and there was no significant difference in the survival time between the two groups (**Figures 1D, E** and **Table 1**). Therefore, A20 would be inactive in HCC cells.

**Table 1:** The association between A20 and the prognosis of HCC patients received Sorafenib.

	A20 mRNA expression		P values
	High (n = 26)	Low (n = 26)	
TTP	10	10	0.85
	6.9-13.1 (M)	8.6-11.4 (M)	
OS	11	10	0.877
	7.0-15.0 (M)	5.7-14.3 (M)	

M, month; TTP, time to progress; OS, overall survival.

Next, the effect of A20 overexpression and A20 knockdown on HCC cells’ sensitivity to sorafenib was examined. As shown in **Figures 1F–H** and **Table 2**, overexpression of A20 did not affect the expression of pro-survival and anti-apoptosis-related factors (**Figure 1F** and **Table 2**). It did not affect the sensitivity of MHCC97-L cells to sorafenib and other agents (**Figures 1G, H** and **Table 2**). Similar results were obtained *via* knockdown of A20 in MHCC97-H cells (**Figures 1I–K** and **Table 3**). A20 is thought to be a tumor suppressor; however, the results were inconsistent; therefore, we needed to explore the mechanism of A20 in HCC further.

**Table 2:** The effect of HSP90 knock down on the sensitivity of MHCC97-H cells to molecular targeted agents.

Agents	Control	siHSP90	BIIB021
	*IC_50_* values (μmol/L)		
Sorafenib	0.56 ± 0.11	0.15 ± 0.05	0.18 ± 0.06
Regorafenib	0.89 ± 0.68	0.06 ± 0.03	0.15 ± 0.03
Lenvatinib	0.42 ± 0.10	0.08 ± 0.04	0.05 ± 0.01
Cabozantinib	0.39 ± 0.21	0.22 ± 0.01	0.19 ± 0.14

**Table 3:** The effect of A20 or HSP90 knockdown on the sensitivity of MHCC97-L cells to molecular targeted agents.

Agents	Control	A20	A20+siHSP90	A20+BIIB021
	*IC_50_* values (μmol/L)			
Sorafenib	1.05 ± 0.25	0.95 ± 0.15	0.15 ± 0.04	0.24 ± 0.11
Regorafenib	0.97 ± 0.08	1.15 ± 0.54	0.10 ± 0.06	0.51 ± 0.22
Lenvatinib	1.21 ± 0.54	1.31 ± 0.39	0.24 ± 0.07	0.13 ± 0.02
Cabozantinib	1.01 ± 0.41	0.93 ± 0.47	0.16 ± 0.13	0.23 ± 0.09

### A20 Formed a Complex With HSP90 and the Complex Could Be Disassociate *via* Downregulation of HSP90

**Figure 2:**
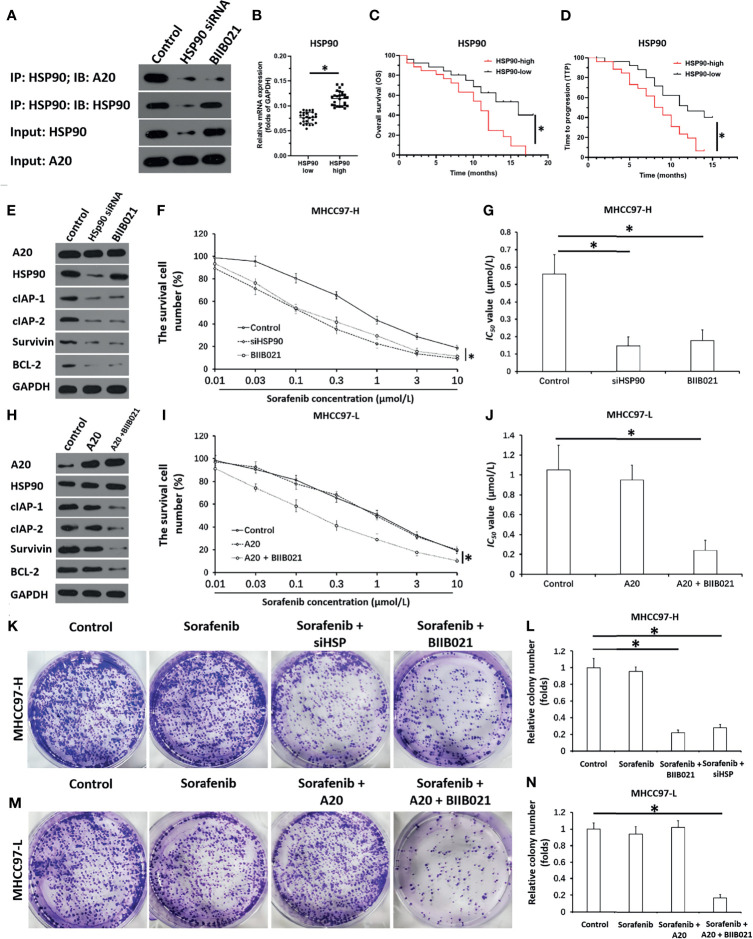
A20 forms complex with HSP90 and inhibits HSP90 rescues the activation of A20. **(A)** The MHCC97-H cells which were transfected with siHSP90 or treated with BIIB021 (the small molecular inhibitor of HSP90) were harvested for the co-Immunoprecipitation. expression level of A20 in HCC or the paired non-tumor tissues was examined by qPCR. **(B)** The expression of HSP90 in HCC clinical specimens was examined by qPCR and the patients were divided into two groups: A20-high group and A20-low group. **(C, D)** The prognosis of two groups’ patients was revealed by the OS (overall survival) **(C)** or the TTP (time to progress) **(D)**. **(E–G)** MHCC97-H was transfected with siHSP or treated with BIIB021. **(E)** The expression level of pro-survival or anti-apoptosis related factors was examined by western blot. **(F, G)** the MHCC97-H cells which were treated with the indicated concentration of Sorafenib and the results were shown as the concentration-effect curve of Sorafenib **(F)** or the *IC_50_* values **(G)**. **(H–J)** MHCC97-L was transfected with control or the A20 vectors or treated with HSP90 inhibitor. **(H)** The expression level of pro-survival or anti-apoptosis related factors was examined by western blot. **(I, J)** The MHCC97-L cells which were transfected with plasmids were treated with the indicated concentration of Sorafenib and the results were shown as the concentration-effect curve of Sorafenib **(H)** or the *IC_50_* values **(J)**. **(K–N)** The MHCC97-H or MHCC97-L cells were transfected with plasmids or treated with Sorafenib and the cells were harvested for the colony formation experiments. The results were shown as the images of colonies or the quantitative results. *P < 0.05.

Next, the mechanism of A20’s effect in HCC cells was examined. As shown in **Figure 2A**, HSP90 interacted with A20 in MHCC97-H cells. Transfection with HSP90’s siRNA and treatment with the HSP90 inhibitor BIIB021 inhibited the interaction between HSP90 and A20. High levels of HSP90 A20 expression with outcome was further examined. Then, the association of HSP90 with HCC patients’ prognosis was also examined. In HCC tissue specimens, according to the median of HSP90 mRNA expression, patients were divided into two groups: the HSP90 high expression group and the HSP90 low expression group (**Figure 2B**). The survival analysis of the two groups was examined. The OS (overall survival) or the TTP (time to progress) of patients with high-level of HSP90 (HSP90-high group) was much shorter than patients with high-level of HSP90 (HSP90-high group) (**Figures 2C, D** and **Table 4**). Therefore, Whether A20 in the HSP90-A20 complex is active may depend on the state of HSP90.

**Table 4:** The association of HSP90 with the prognosis of HCC patients receive Sorafenib.

	HSP90 mRNA expression		P values
	High (n = 26)	Low (n = 26)	
TTP	8	12	0.01
	6.0-10.0 (M)	7.2-16.8 (M)	
OS	11	16	0.02
	7.7-14.3 (M)	9.4-22.6 (M)	

M, month; TTP, time to progress; OS, overall survival.

### Downregulation of HSP90 *via* Its siRNA or Small Molecular Inhibitor Enhances the *In Vitro* Sensitivity of HCC Cells to Macular Targeted Agents

The effect of the disassociation of the A20/HSP90 complex on HCC cells’ sensitivity to sorafenib was examined. As shown in **Figures 2E–G** and **Table 3**, in MHCC97-H cells with high level of A20, knockdown of HSP90 *via* its siRNA or small molecular did not affect the expression of A20 but decreased the expression of pro-survival and anti-apoptosis-related factors (**Figures 2E–G** and **Table 3**) and enhanced the sensitivity of MHCC97-H cells to molecular targeting agents. Moreover, in MHCC97-L cells with low level of endogenous A20, overexpression of A20 alone did not affect the sensitivity of MHCC97-L cells to sorafenib and other agents or the expression of pro-survival or anti-apoptosis related factors (**Figures 2H–J** and **Table 2**), however, overexpression of A20 combined with HSP90 inhibiter not only enhanced the sensitivity of MHCC97-L cells to sorafenib and other agents but also inhibited the expression of pro-survival or anti-apoptosis related factors (**Figures 2H–J** and **Table 2**). The results from cellular survival examination (the MTT experiments) was confirmed by colony formation experiments (**Figures 2K–N**). Therefore, downregulation of HSP90 *via* its siRNA or small molecular inhibitor enhances the *in vitro* sensitivity of HCC cells to macular targeted agents

### Knockdown of HSP90 Enhanced the *In Vivo* Antitumor Activity of Sorafenib and Repressed the Pro-Survival and Anti-Apoptosis-Related Factors in Subcutaneous Tumors

**Figure 3:**
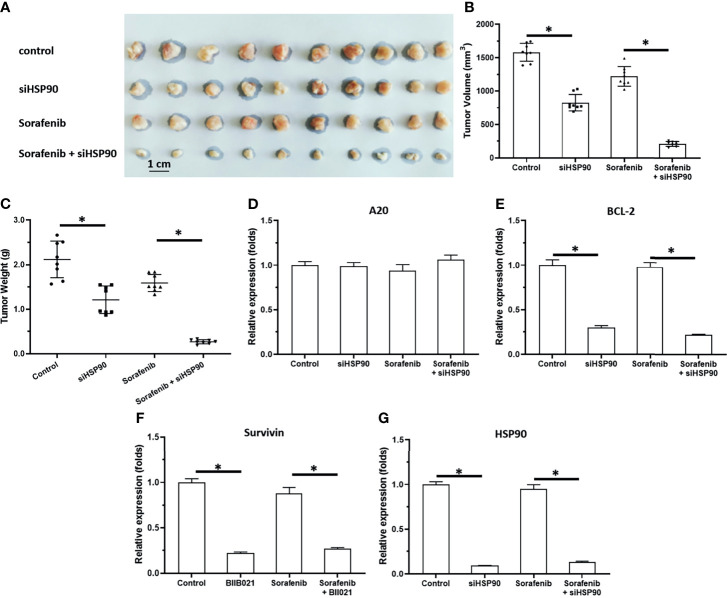
Knockdown of HSP90 in MHCC97-H cells *via* siRNA enhances the antitumor effect of Sorafenib on the subcutaneous growth of MHCC97-H cells in nude mice. MHCC97-H cells were transfected with control or siHSP90. HCC97-H cells were injected into the nude mice to form the subcutaneous tumors. Mice were received Sorafenib *via* oral administration. The results were shown as the images of tumor tissues **(A)**, tumor volumes **(B)**, tumor weights **(C)** or the expression level of pro-survival or anti-apoptosis related factors in the tumor tissues **(D–G)**. *P < 0.05.

**Figure 4:**
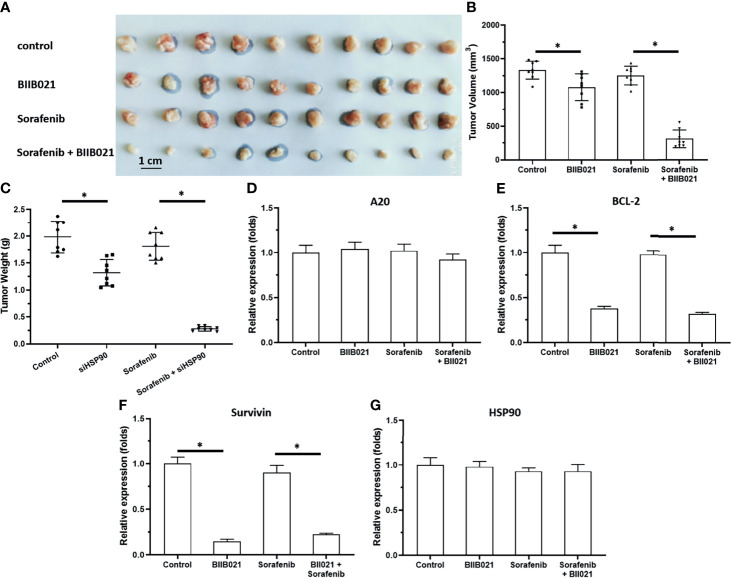
Knockdown of HSP90 in MHCC97-H cells *via* BIIB021 enhances the antitumor effect of Sorafenib on the subcutaneous growth of MHCC97-H cells in nude mice. MHCC97-H cells were injected into the nude mice to form the subcutaneous tumors. Mice were received Sorafenib or BIIB021 *via* oral administration. The results were shown as the images of tumor tissues **(A)**, tumor volumes **(B)**, tumor weights **(C)** or the expression level of pro-survival or anti-apoptosis related factors in the tumor tissues **(D–G)**. *P < 0.05.

To further examine the effect of HSP90, the subcutaneous tumor model was used. As shown in **Figures 3**, **4**, MHCC97-H cells formed subcutaneous tumors. Oral administration of 0.5 mg/kg sorafenib did not significantly affect the subcutaneous growth of HCC cells in nude mice. Knockdown of HSP90 *via* its siRNA and oral administration of HSP90 enhanced the sensitivity of HCC cells to sorafenib. It repressed the expression of NF-κB downstream pro-survival and anti-apoptosis-related factors (**Figures 3** and **4**). These findings suggest that the inhibitory effect of A20 was rescued in the presence of HSP90 siRNA and HSP90 inhibitor.

### Knockdown of HSP90 Enhanced the *In Vivo* Antitumor Activity of Sorafenib and Repressed the Pro-Survival and Anti-Apoptosis-Related Factors in the Intrahepatic Tumor Model

**Figure 5:**
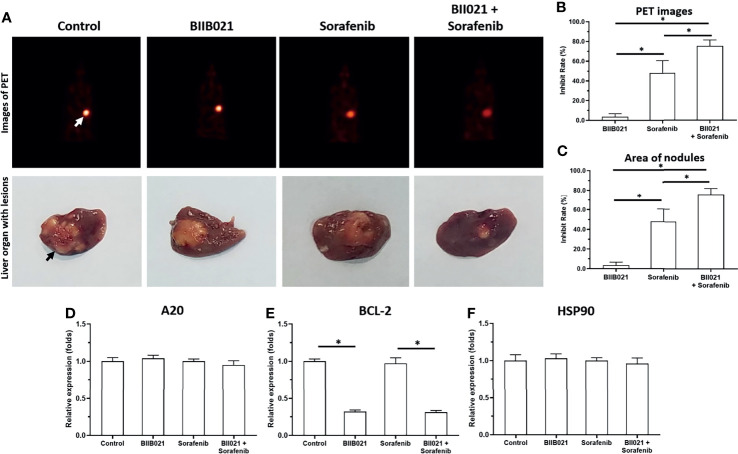
Knockdown of HSP90 in MHCC97-H cells *via* BIIB021 enhances the antitumor effect of Sorafenib on the intrahepatic growth of MHCC97-H cells in nude mice. MHCC97-H cells were injected into the liver organs of nude mice to form the intrahepatic nodules/lesions. Mice were received Sorafenib or BIIB021 *via* oral administration. The results were shown as the images of microPET or liver organs with lesions/nodules **(A)**, the quantitative results **(B, C)** or the expression level of pro-survival or anti-apoptosis related factors in the tumor tissues **(D–F)**. *P < 0.05. The white arrow indicates the image of the nude mouse liver region in the microPET images **(A)** and the black arrow indicates the HCC lesions/nodules formed by the MHCC97-H cells in the nude mouse liver organs **(A)**.

The previous results focused on the subcutaneous growth of HCC cells. The *in vivo* growth of HCC cells was further examined using an intrahepatic tumor model. As shown in **Figure 5**, MHCC97-H cells could form the intrahepatic lesions or nodules in nude mice’s liver organs and the intrahepatic growth of MHCC97-H cells were measured by the microPET or the images of liver organs. Treatment of 1mg/kg Sorafenib could inhibit the intrahepatic growth of MHCC97-H cells. Treatment of BIIB021, the small molecular inhibitor of HSP90, enhanced the antitumor effect of Sorafenib on MHCC97-H’s intrahepatic growth. The results were shown as the images of liver organs with nodules (**Figure 5A**) or the quantitative results (**Figures 5B, C**). The effect of Sorafenib or BIIB021 on MHCC97-H cells was confirmed by the qPCR examining the pro-survival or anti-apoptosis related factors in lesions tissues (D-F). Therefore, the Knockdown of HSP90 enhanced the *in vivo* antitumor activity of sorafenib and repressed the pro-survival and anti-apoptosis-related factors in the intrahepatic tumor model.

## Discussion

**Figure 6:**
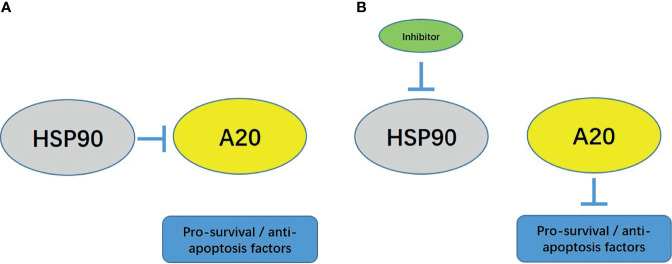
The proposal model of the presence work. **(A)** In HCC cells, HSP90 forms a complex with A20, and A20 cannot function at this time. **(B)** Using small molecule inhibitors to act on HCC cells can inhibit the activity of HSP90 and dissociate the complex between HSP90 and A20. A20 recovers at this time.

Although many advantages have been achieved, the treatment of advanced HCC faces considerable obstacles (43). First, the one-third, two-thirds, and whole liver can only accept 90, 47, and 31 Gy doses of IR, respectively; however, these doses of IR are only partial doses of the HCC-control dose. Radio-resistance limits the application of radiotherapy for HCC treatment (6). Moreover, local therapeutic strategies, such as radiofrequency ablation, transhepatic artery chemoembolization, and cryoablation are invasive therapies for advanced HCC; the associated recurrence rates after local treatment cannot be ignored (44, 45). Therefore, it critical to identify targeted molecular therapies to achieve safer and more effective strategies. Several studies provided molecular clues that the pro-survival and anti-apoptotic response to NF-κB would be potential targets (46, 47). Based on the evidence that zinc finger protein A20 functioned as a negative regulator of NF-κB, overexpression of A20 and enhancing the activation of A20 could enhance HCC cells’ sensitivity to molecularly targeted agents *via* restricting the activation of the NF-κB pathway (6). However, the function of A20 in HCC has been rarely reported, and there are no universal roles regarding A20’s function. Liu et al. suggested that A20 was a tumor suppressor in HCC that enhanced HCC cells’ sensitivity to ^60^Co-γ IR (6). Chen et al. found that overexpression of A20 repressed the proliferation and metastasis of HCC cells *via* inhibiting the expression of Twist1 with higher expression levels in HCC tissues and cell lines than in non-tumor control (48). Other investigators reported opposite trends. Dong et al., Wang et al. and Wang et al. showed that A20 was a positive regulator of HCC cell survival and proliferation (49–51), and knockdown of A20 attenuated the proliferation and metastasis of HCC cells and protected the cells from injury induced by TNFα treatment (49–51). In the present study, the expression of A20 in HCC tissues was higher than in non-tumor tissues, but A20 itself may not affect the activity of the pro-survival/anti-apoptosis related downstream factors of NF-κB in HCC cells (overexpression and knockdown of A20 expression did not affect the pro-survival/anti-apoptosis related downstream factors of NF-κB (**Figure 6**). The expression of pro-survival and anti-apoptotic factors in downstream cells of NF-κB) suggests that A20 may lose activity in HCC cells

Furthermore, we found that HSP90 and A20 form a complex in HCC cells to inhibit the activity of A20. The use of HSP90 siRNA and HSP90 small molecule inhibitors can inhibit HSP90 and A20 complexes’ formation and finally release A20 from the complex. Restore activity, and finally down-regulate the expression level of NF-κB downstream related factors. The expression of A20 in HCC tissues does not correlate with the prognosis of patients, while HSP90 is highly expressed in HCC tissues, and the expression level of HSP90 is negatively correlated with the prognosis of patients.

The HSP90 (heat shock proteins 90) is present at low levels under normal conditions; however, these levels are significantly elevated in the presence and injury in response to cellular stresses (52). Previously, HSP90 was considered an essential molecular chaperone that participates in physical processes, including protein homeostasis, cellular apoptosis, migration/invasion, and cellular signaling transduction (53). Studies have provided clues that HSPs functions as an essential regulator of HCC and overexpression of HSP90 (54). HSP90 is often associated with poor outcome and antitumor treatment resistance (55). Knockdown of HSP90 activation using small molecular inhibitors such as 5-aryl-3-thiophen-2-yl-1H-pyrazoles and AUY922 (luminespib) attenuated the proliferation of HCC cells (56–58). Mechanically, the oncogenic effect of HSP90 is often associated with some oncogene or pro-oncogene, e.g., c-MYC (59). Our findings suggest that HSP90 interacts with A20, while A20 in the A20-HSP90 complex may not be active. Treating cells with inhibitors of HSP90 induces the dissociation of the HSP90-A20 complex, and A20 was released. The activity of A20 enhancing the sensitivity of HCC cells to molecularly targeted drugs was rescued. The results of this study expand our understanding of HSP90 regulation of HCC cells.

It is worth mentioning that the cellular stress response mechanism has attracted considerable attention (60). For example, the cellular injury response mechanism represented by Notch is closely related to the proliferation, metastasis, and invasion of malignant tumor cells such as HCC, and it induced resistance to anti-tumor treatments (61, 62). Cytotoxic chemotherapy drugs, molecularly targeted drugs, IR, and thermal ablation can be used as damage/stress factors to cells to induce activation of the Notch pathway (63, 64). The activated Notch pathway promotes survival by inducing pro-survival and anti-apoptosis-related factors, protecting cells, and ultimately inducing cells’ resistance to anti-tumor treatments (65, 66). The stress response is the primary function of HSP90 (67, 68). The occurrence and progression of HCC are closely related to inflammation (69). Under repeated stimulation of liver injury induced by the hepatitis B virus, liver cells eventually become cancerous (69, 70). HSP90 is closely related to the hepatitis B virus. The high expression of HSP90 in HCC resulted from the induction of stress factors such as inflammation and liver damage during the occurrence and progression of HCC (71). A20 is thought to be a tumor suppressor; it was initially discovered as an inhibitor of inflammatory responses and other stress factors (72). Taken together, these findings suggest that high expression levels of A20 in HCC may represent the body’s protective effect against liver damage and stress responses. However, high expression levels of HSP90 in HCC cells eventually cause A20 inactivation by forming a complex with HSP90. This study’s results provide a possible mechanism for the inconsistency of current A20-related research: A20 can be used as a tumor suppressor; however, A20’s role depends on whether HSP90 is aberrantly expressed. The cellular stress response process is closely related to the tumor tissue microenvironment, and the tumor tissue microenvironment is closely related to the epithelial-mesenchymal transition of malignant tumor cells (73–76). Future studies will focus on the relationship between HSP90 and epithelial-mesenchymal transition.

## Conclusion

A20 and HSP90 form a complex in HCC cells, and A20 loses its activity. The HSP90-A20 complex dissociated and released A20, which rescues the activity of A20 and enhances the sensitivity of HCC cells to molecularly targeted agents.

## Data Availability Statement

The original contributions presented in the study are included in the article/supplementary material. Further inquiries can be directed to the corresponding authors.

## Ethics Statement

The studies involving human participants were reviewed and approved by ethics committee of the Fifth Medical Center of Chinese PLA General Hospital. The patients/participants provided their written informed consent to participate in this study. The animal study was reviewed and approved by the animal ethic committee of the fifth medical center, the PLA General Hospital of China.

## Author Contributions

L-jS, W-mL, and S-jX: Conceptualization, Methodology, Software. S-jX and W-mL: Data curation, Writing- Original draft preparation. L-jS, Q-yJ, H-wS, and Y-tC: Visualization, Investigation. L-jS and JZ: Supervision. JZ, Q-yJ, and H-wS: Software, Validation. L-jS, S-jX, W-mL, Q-yJ and H-wS: Writing- Reviewing and Editing. All authors contributed to the article and approved the submitted version.

## Funding

This work was supported by the 13th Five-Year National Science and Technology Major Project for Infectious Diseases (Severe Hepatitis B (Liver Failure) Research on New Technologies and Programs for Clinical Treatment) (Grant number 2017ZX10203201-004).

## Conflict of Interest

The authors declare that the research was conducted in the absence of any commercial or financial relationships that could be construed as a potential conflict of interest.

## Publisher’s Note

All claims expressed in this article are solely those of the authors and do not necessarily represent those of their affiliated organizations, or those of the publisher, the editors and the reviewers. Any product that may be evaluated in this article, or claim that may be made by its manufacturer, is not guaranteed or endorsed by the publisher.
